# Interleukin‐11 regulates the fate of adipose‐derived mesenchymal stem cells via STAT3 signalling pathways

**DOI:** 10.1111/cpr.12771

**Published:** 2020-04-09

**Authors:** Wenlong Yang, Shuning Zhang, Tiantong Ou, Hao Jiang, Daile Jia, Zhiyong Qi, Yunzeng Zou, Juying Qian, Aijun Sun, Junbo Ge

**Affiliations:** ^1^ Department of Cardiology Zhongshan Hospital Fudan University Shanghai China; ^2^ Shanghai Institute of Cardiovascular Diseases Shanghai Cardiovascular Medical Center Institute of Pan‐vascular Medicine Fudan University Shanghai China; ^3^ Institute of Biomedical Sciences Fudan University Shanghai China

**Keywords:** adipose‐derived mesenchymal stem cells, cell therapy, interleukin‐11, limb ischaemia

## Abstract

**Objective:**

Adipose‐derived mesenchymal stem cells (ADSCs) offer great promise as cell therapy for ischaemic diseases. Due to their poor survival in the ischaemic environment, the therapeutic efficacy of ADSCs is still relatively low. Interleukin‐11 (IL‐11) has been shown to play a key role in promoting cell proliferation and protecting cells from oxidative stress injury. The aim of this study was to determine whether IL‐11 could improve therapeutic efficacy of ADSCs in ischaemic diseases.

**Methods and Results:**

ADSCs were prepared from inguinal subcutaneous adipose tissue and exposed to hypoxic environment. The protein expression of IL‐11 was decreased after hypoxic treatment. In addition, ADSCs viability was increased after IL‐11 treatment under hypoxia. Moreover, IL‐11 enhanced ADSCs viability in a dose‐dependent manner under normoxia. Importantly, IL‐11 promoted ADSCs proliferation and migration and protected ADSCs against hydrogen peroxide‐induced cellular death. Notably, IL‐11 enhanced ADSCs proliferation and migration, also promoted cell survival and apoptosis resistance by STAT3 signalling. In vivo, mice were subjected to limb ischaemia and treated with IL‐11 overexpression ADSCs and control ADSCs. IL‐11 overexpression ADSCs improved perfusion recovery in the ischaemic muscles.

**Conclusions:**

We provide the evidence that IL‐11 promoted ADSCs proliferation, stimulated ADSCs migration and attenuated ADSCs apoptosis by activation of STAT3 signalling. These results suggest that IL‐11 facilitated ADSCs engraftment in ischaemic tissue, thereby enhanced ADSCs therapeutic efficacy.

## 1 INTRODUCTION

Mesenchymal stem cells (MSCs) are multipotent stromal cells from many tissue sources, including the bone marrow, adipose tissue, umbilical cord and other adult tissues.^1^ MSCs are capable of differentiating into multiple lineages of tissues, such as vasculature, fat and cartilage, under lineage‐specific culture conditions.^2^, ^3^ Multiple studies utilizing MSCs have been conducted, being considered a promising treatment for ischaemic tissue injuries, such as hindlimb ischaemia and myocardial infarction.^4^, ^5^, ^6^ Due to the simplicity and reproducibility of the isolation process, adipose tissue has become an attractive source of MSCs for regenerative medicine.^7^, ^8^ In recent years, multiple studies have been performed to use adipose‐derived mesenchymal stem cells (ADSCs) for the revascularization and tissue repair of ischaemic tissues. The engrafted ADSCs can favourably promote neovascularization of ischaemic limbs and effectively improve cardiac function after myocardial infarction in animal models.^9^, ^10^ The benefits of ADSCs transplantation may be attributed to their multilineage differentiation ability within ischaemic tissues and paracrine secretion of growth factors produced by ADSCs, such as stromal cell‐derived factor‐1 and vascular endothelial growth factor.^2^, ^11^, ^12^ However, the beneficial effects observed in animal experiment have not been fully translated to patients with ischaemic injuries. The poor survival of implanted MSCs in the ischaemic environment is the main limitation of the therapeutic potential of MSCs.^1^, ^13^ Therefore, overcoming this limitation by promoting the survival of MSCs or improving the local environment may improve the efficacy of MSCs therapy for ischaemic disease.^14^

The interleukin‐11 (IL‐11) is a member of the interleukin‐6 cytokine family due to their common feature of using the type I cytokine receptor glycoprotein 130 as the β‐subunit in their multimeric receptor complexes.^15^ In 1990, IL‐11 was identified from the supernatants of immortalized primate bone marrow stromal cells with the activity of plasmacytoma stimulatory.^16^, ^17^ During normal homeostasis, IL‐11 expression levels are usually low and therefore difficult to detect. However, it is increasingly clear that IL‐11 is produced by cells within the heart, central nervous system and gastrointestinal tract.^18^, ^19^, ^20^ In clinical, IL‐11 has been used for the prevention of chemotherapy‐induced thrombocytopenia due to its megakaryocytopoiesis activity in patients with non‐myeloid malignancies.^21^ In addition, previous studies have suggested that IL‑11/receptor interaction leads to activation of JAK/STAT3 signalling pathway.^22^ Studies have shown that IL‐11 prevents skeletal myoblasts apoptosis and endothelial cell injury under oxidant stress via STAT3 signalling pathway.^23^, ^24^ Furthermore, research has shown that the expression of IL‐11 significantly decreases during cerebral ischaemia‐reperfusion injury, and IL‐11 treatment improved neurological function and cerebral infarct volume.^25^ It is also shown that IL‐11 is beneficial for ischaemia‐reperfusion injury of kidneys, heart and intestines.^26^, ^27^, ^28^

Because IL‐11 plays a key role in promotion of megakaryocytopoiesis and thrombopoiesis, recombinant human IL‐11 has been extensively used in patients with good clinical efficacy and safety parameters. Thus, we performed a pre‐clinical study to explore the role of IL‐11 as a potential pharmacological agent for improving the efficacy of stem cell therapy using a hindlimb ischaemia mouse model. In this study, we examined whether IL‐11 treatment improves ADSCs therapeutic efficiency in hindlimb ischaemia tissues and explored the underlying mechanisms of IL‐11 in regulating the function of ADSCs under oxidative stress.

## 2 MATERIALS AND METHODS

### 2.1 Animal study protocol

C57BL/6 male mice (8‐week‐old) were purchased from the Experimental Animal Center of Fudan University, China. Mice were housed in a temperature‐controlled environment with 12 hour/12 hour light/dark cycles. Mice were randomly divided into three groups (n = 6): Mice were performed hindlimb ischaemia operation (hindlimb ischaemia group, HI), hindlimb ischaemia operation with control ADSCs transplantation (HI + ADSCs^con^ group) or with IL‐11 overexpression ADSCs transplantation (HI + ADSCs^IL‐11^). The Experimental Animal Ethic Committee of Fudan University approved the animal research protocol. All the animal procedures were performed in accordance with the Guiding Principles in the Use and Care of Animals (NIH Publication No. 85‐23, revised 1996).

### 2.2 Isolation and culture of mouse ADSCs

Adipose‐derived mesenchymal stem cells were isolated from inguinal subcutaneous adipose tissue as previously described.^7^ Briefly, adipose tissues were cut into small pieces in phosphate‐buffered saline (PBS) on ice. The minced tissue was then digested with 1 mg/mL Type 1 collagenase (Worthington‐Biochem, LS004196) at 37°C for 1 hour. The digested tissue was filtered through a 70 µm mesh (Corning, 431751) to remove tissue debris. The cell suspension was then centrifuged at 600 *g* for 15 minutes to remove collagenase. The cell pellet was plated in 100 mm dishes and incubated with 1× lysis buffer (Beyotime, C3702) at room temperature for 10 minutes to lyse red blood cells. After lysis of red blood cells, the pellets were maintained in mouse ADSCs complete medium (Cyagen, MUBMD‐90011) at 37°C in an atmosphere with 5% CO_2_. Medium was changed after 24 hours and then every second day. ADSCs were used for subsequent experiments from the second passage. Recombinant mouse IL‐11 protein (R&D Systems, 418‐ML), Stattic (Selleck, S7024) and anti‐IL‐11Rα (R&D systems, AF490) were used to treat ADSCs.

### 2.3 Flow cytometry

Adipose‐derived mesenchymal stem cells were digested by trypsinization and washed with PBS. For flow cytometry, 1 × 10^6^ ADSCs were stained with fluorescent antibodies at room temperature for 1 hour in PBS. The following antibodies and their non‐specific negative isotype controls were employed: FITC‐CD29 (Invitrogen, 11‐0291‐80), FITC‐CD105 (Invitrogen, MA5‐17945), FITC‐Sca‐1 (Invitrogen, 11‐5981‐81) and PE‐CD45 (BD Pharmingen, 553081). After incubation, cells were washed three times with PBS and centrifuged at 300 *g* for 10 minutes, and cells were then resuspended in PBS for flow cytometry. Surface marker expression was evaluated via flow cytometry (BD LSR Fortessa™). FlowJo software was used for data analysis.

### 2.4 Adipogenesis and osteogenesis

Adipogenic and osteogenic differentiation of ADSCs were performed as previously reported.^7^ For adipogenesis, cells were incubated in adipogenic medium (Cyagen, MUBMD‐90031) for 21 days. The medium was changed every three days. Adipogenesis was assessed by Oil Red O solution to stain lipids. For osteogenesis, cells were incubated in osteogenic medium (Cyagen, MUBMD‐90021) for 21 days. The medium was changed every three days. Osteogenesis was evaluated by alizarin red staining solution.

### 2.5 Mouse hindlimb ischaemia model and cell transplantation

Hindlimb ischaemia model was established as previously described.^29^ In brief, mice were anaesthetized with pentobarbital sodium (50 mg/kg) intraperitoneally. The femoral artery was separated from the femoral nerve and vein, and then, artery was ligated and excised. One day after the surgery, ADSCs (1 × 10^6^) suspended in 100 µL PBS or equal PBS was injected intramuscularly into the ischaemic hindlimb in three different sites. Hindlimb perfusion was evaluated by laser Doppler perfusion imaging (PeriScan PIM 3 system, Perimed) at 7 and 14 days. PIMsoft Software (Perimed med) was used to quantify perfusion ratio of ischaemic limb versus non‐ischaemic limb by averaging relative units of flux.

### 2.6 Masson's trichrome staining

Hindlimb fibrosis was evaluated by staining with Masson Trichrome reagent (Yeasen, 60532ES58). Tissues were harvested and then fixed in formalin. Sections (5 μm thick) were prepared for Masson's trichrome staining according to manufacturer's instructions. Fibrosis was measured via inverted optical microscope (ZEISS Group).

### 2.7 Immunofluorescence

The isolated muscular tissues were embedded in OCT, and the frozen sections were prepared and fixed in 4% paraformaldehyde. Sections were incubated with anti‐GFP (CST, 2956) antibodies overnight at 4°C and then incubated with goat anti‐rabbit IgG, Alexa Fluor 488 (Thermo Fisher, A‐11008) for 30 minutes at 37°C. The nuclei were stained with DAPI for 10 minutes at room temperature. Sections were visualized using inverted optical microscope (ZEISS Group). The GFP‐positive cells were counted with Image J software (National Institutes of Health, Bethesda, MD, USA).

### 2.8 Cell viability assay

The Cell Counting Kit‐8 (CCK‐8) assay (Beyotime, C0037) was performed to determine cell viability. Briefly, ADSCs were seeded into a 96‐well plate and treated with IL‐11or not. After 24 hours, 10 μL of CCK‐8 solution was added to each well and incubated at 37°C for 2 hours. The absorbance at a wavelength of 450 nm was read using microplate reader (SpectraMax^®^ M5, Molecular Devices).

### 2.9 Cell proliferation assay

The second passage ADSCs were seeded in a 12‐well plate and treated with IL‐11 or not after 24 hours. Then, the cells were stained using the BeyoClick™ EdU‐488 kit (beyotime, C0071S) according to manufacturer's protocol and observed by inverted fluorescence microscope (ZEISS Group). The number of positive nuclei/the total nuclei were counted using Image J software (National Institutes of Health).

### 2.10 Cell apoptosis assessment

Apoptosis of ADSCs was detected by terminal deoxynucleotidyl transferase‐mediated dUTP nick‐end labelling (TUNEL) staining. TUNEL Detection Kit Fluorescein (Beyotime, C1088) was employed to determined apoptosis of ADSCs according to manufacturer's protocol. The number of TUNEL positive nuclei/the total nuclei were counted to calculate the index of apoptosis.

### 2.11 Transwell assay

The Costar^®^ Transwell^®^Insert (8 µm, Corning 3422) was employed in migration assay as previously described.^30^ ADSCs were seeded in the upper chamber in serum‐free medium. The complete medium containing IL‐11 or not were added in the bottom wells. 0.1% crystal violet was used to stain cells in the upper chamber. Cells in the bottom well were counted using inverted optical microscope (ZEISS Group).

### 2.12 Cell transfection

1 × 10^6^ ADSCs were seeded into 6‐well plates. The medium was removed after 24 hours of culture and then replaced with serum‐free DMEM containing 10 μL of pLenti‐EF1a‐EGFP‐P2A‐Puro‐CMV‐IL11‐3Flag (IL‐11 overexpression group) or pLenti‐EF1a‐EGFP‐P2A‐Puro‐CMV‐MCS‐3Flag (control group) for a further 24 hours. After transfection with pLenti‐EF1a‐EGFP‐P2A‐Puro‐CMV‐MCS‐3Flag for 48 hours, the efficiency of transfection was determined by fluorescence microscopy. Alterations of protein were corroborated by Western blot.

### 2.13 Western blotting

Proteins were isolated from ADSCs lysates, added the same amount of protein to the sodium dodecyl sulphate polyacrylamide gel and transferred to a PVDF membrane. The membranes were incubated with primary antibody at 4℃ overnight and added secondary antibody next day. Then, the membrane was visualized with enhanced chemiluminescence and quantified by densitometry. The primary antibodies included mTOR (7C10) Rabbit mAb (CST, 2983) (1:1,000), Phospho‐mTOR (Ser2448) (D9C2) XP^®^ Rabbit mAb (CST, 5536, 1:1,000), p44/42 MAPK (Erk1/2) (137F5) Rabbit mAb (CST, 4695, 1:1,000), Phospho‐p44/42 MAPK (Erk1/2) (Thr202/Tyr204) Antibody (CST, 9101, 1:1000), Stat3 (D1B2J) Rabbit mAb (CST, 30835, 1:1000) and Phospho‐Stat3 (Tyr705) (D3A7) XP^®^ Rabbit mAb (CST, 9145, 1:1000).

### 2.14 Statistical analysis

All data are presented as means ± standard deviation. Student's *t* test (two groups) or one‐way ANOVA followed by Dunnett's post hoc test was employed for comparisons between the groups using the GraphPad Prism Software Version 5.9. Values of *P* < .05 were considered statistically significant.

## 3 RESULTS

### 3.1 IL‐11 promoted ADSCs growth in a dose‐dependent manner

**Figure 1:**
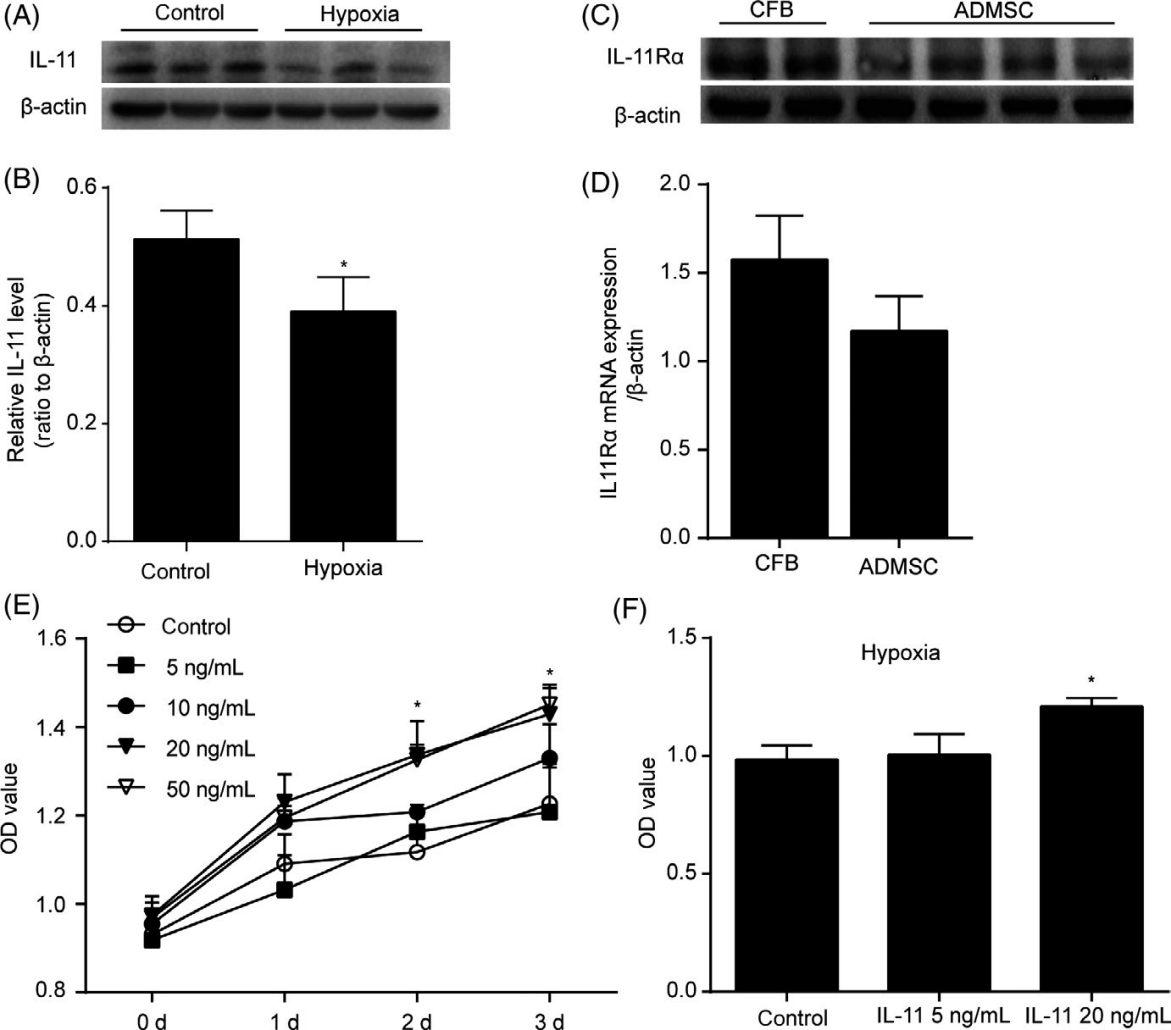
IL‐11 promoted ADSCs growth in a concentration‐dependent manner. A and B, Western blots and quantification protein expression of IL‐11 under hypoxia. *P < .05 vs control. Unpaired Student's *t* test for statistical analyses; C and D, Western blots and quantification protein expression of IL‐11Rα in ADSCs and CFB; E, ADSCs growth curves. CCK‐8 assay was performed after different concentrations of IL‐11 or vehicle treatment at 0, 1, 2 and 3 days. *P < .05 vs control. Two‐way ANOVA followed by Dunnett's post hoc test for statistical analyses. F, CCK‐8 assay was performed 24 h after IL‐11 or vehicle treatment under hypoxia. *P < .05 vs control. Two‐way ANOVA followed by Dunnett's post hoc test for statistical analyses. (At least three independent experiments were performed)

The second passage ADSCs were used for detecting the stroma‐associated markers and differentiation potential. ADSCs expressed mesenchymal stem cells markers CD29 (98.9 ± 0.8%), CD105 (91.6 ± 1.1%) and Sca‐1 (77.5% ± 1.7%), but few haematopoietic lineage marker CD45 (0.1 ± 0.2%) (Figure S1A,B). The adipogenic and osteoblastic potential of ADSCs demonstrated their pluripotency (Figure S1C). First, we examined the expression of IL‐11 under hypoxia. Western blot analysis demonstrated that the expression of IL‐11 was decreased under hypoxia compared with the control group (*P* < .05, Figure 1A,B). To explore whether IL‐11 receptor was expressed in ADSCs, we examined the expression of IL‐11 receptor using cardiac fibroblasts as a positive control.^18^ The results indicated that ADSCs expressed extensive amount of IL‐11 receptor (Figure 1C,D). In addition, ADSCs were treated with different concentrations of IL‐11. The CCK‐8 assay demonstrated that IL‐11 promoted cell growth in a dose‐dependent manner (*P* < .05, Figure 1E). According to the results of CCK‐8 assay, we chose 20 ng/mL of IL‐11 for follow‐up experiments. Furthermore, IL‐11 enhanced ADSCs viability under hypoxia (*P* < .05, Figure 1F).

### 3.2 IL‐11 promoted ADSCs proliferation, migration and protected ADSCs from hydrogen peroxide‐induced cell apoptosis

**Figure 2:**
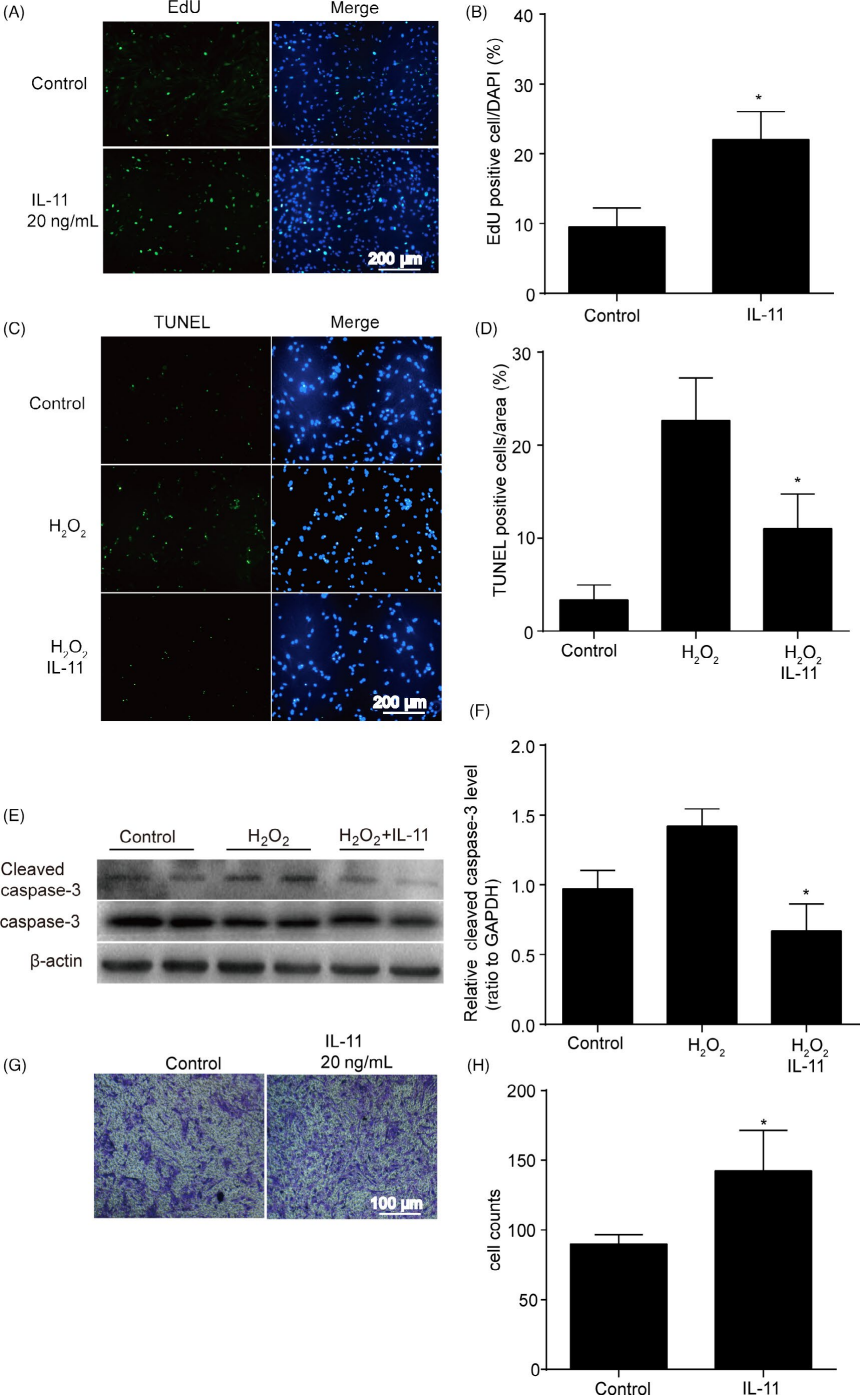
IL‐11 promoted ADSCs proliferation, migration and protected ADSCs from hydrogen peroxide‐induced cell apoptosis. A and B, Representative images and quantitative analysis of ADSCs proliferation. EdU assay was performed 24 h after IL‐11 (20 ng/mL) or vehicle treatment. *P < .05 vs control. Unpaired Student's *t *test for statistical analyses; C and D, representative images and quantitative analysis of ADSCs apoptosis. TUNEL staining assay was performed 24 h after IL‐11 (20 ng/mL) or vehicle treatment. H_2_O_2_: 200 μmol/L, 24 h. *P < .05 vs H_2_O_2_ + vehicle. Two‐way ANOVA followed by Dunnett's post hoc test for statistical analyses; E and F, Western blots and quantification protein expression of cleaved caspase‐3 in ADSCs with H_2_O_2_ or IL‐11 + H_2_O_2_ treatment. IL‐11:20 ng/mL, 24 h. H_2_O_2_: 200 μmol/L, 24 h. *P < .05 vs control. Unpaired Student's *t *test for statistical analyses; G and H, Cell migration analysis assessed by transwell assay 12 h after IL‐11 (20 ng/mL) or vehicle treatment. *P < .05 vs control. Unpaired Student's* t *test for statistical analyses; (n = 5 different fields. (At least three independent experiments were performed)

To determine whether IL‐11 regulated ADSCs function, we examined the proliferation, migration and apoptosis of ADSCs treated with or without IL‐11 in vitro. The results indicated IL‐11 increased ADSCs proliferation after 24 hours treatment compared with vehicle (*P* < .05, Figure 2A,B). ADSCs with H_2_O_2_ treatment increased the expression of cleaved caspase‐3 and TUNEL positive nuclei. IL‐11 treatment significantly attenuated these markers of apoptosis (*P* < .05, Figure 2C‐F). The transwell assays suggested that ADSCs showed a markedly enhanced migratory capacity after IL‐11 treatment (*P* < .05, Figure 2G,H). In addition, IL‐11 has no effect on the VEGF and FGF2 secretion of ADSCs (Figure S2). These results demonstrated that IL‐11 promoted ADSCs proliferation, migration and survival in vitro.

### 3.3 IL‐11 activated the STAT3 signalling pathway

**Figure 3:**
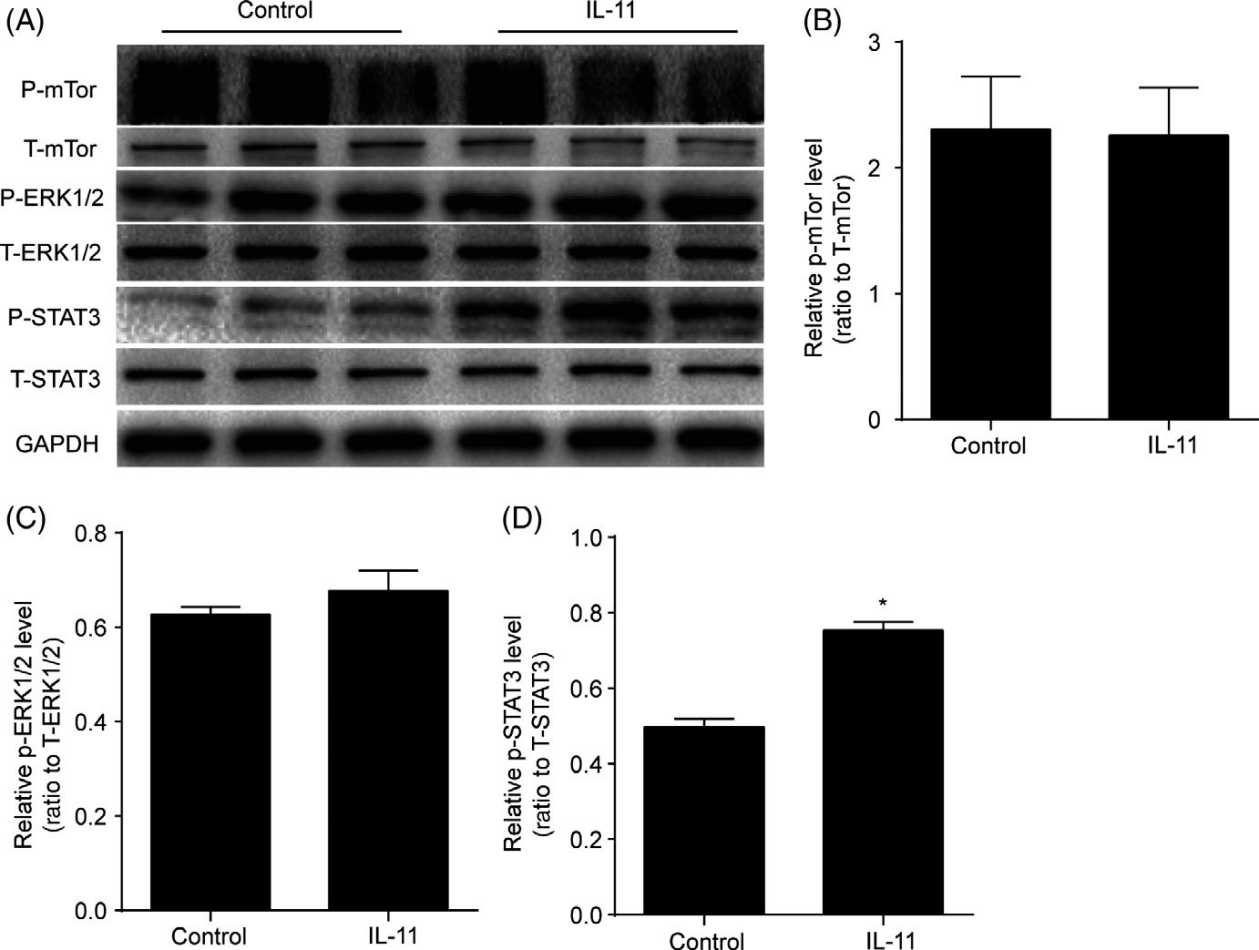
IL‐11 activated the STAT3 signalling pathway. A, Western blot analysis of P‐mTor, T‐mTor, P‐ERK1/2, T‐ERK1/2, P‐STAT3 and T‐STAT3 in ADSCs in response to IL‐11 (20 ng/mL) treatment. B‐D, Quantitative analysis of the expression of P‐mTor, P‐ERK1/2 and P‐STAT3 in ADSCs in response to IL‐11 (20 ng/mL) treatment. *P < .05 vs control. Unpaired Student's *t* test for statistical analyses; (At least three independent experiments were performed)

To determine the molecular mechanisms underlying the effects of IL‐11 upon ADSCs, we examined the expression of P‐STAT3, P‐ERK1/2 and P‐mTOR in ADSCs with IL‐11 treatment. Western blot analysis revealed that only the expression of P‐STAT3 was significantly up‐regulated after 24 hours of IL‐11 treatment (*P* < .05, Figure 3A‐D). These results suggested that IL‐11 may regulate the function of ADSCs via STAT3 signalling pathway.

### 3.4 IL‐11 induced ADSCs proliferation, migration and anti‐apoptotic effects via STAT3 signalling pathway

**Figure 4:**
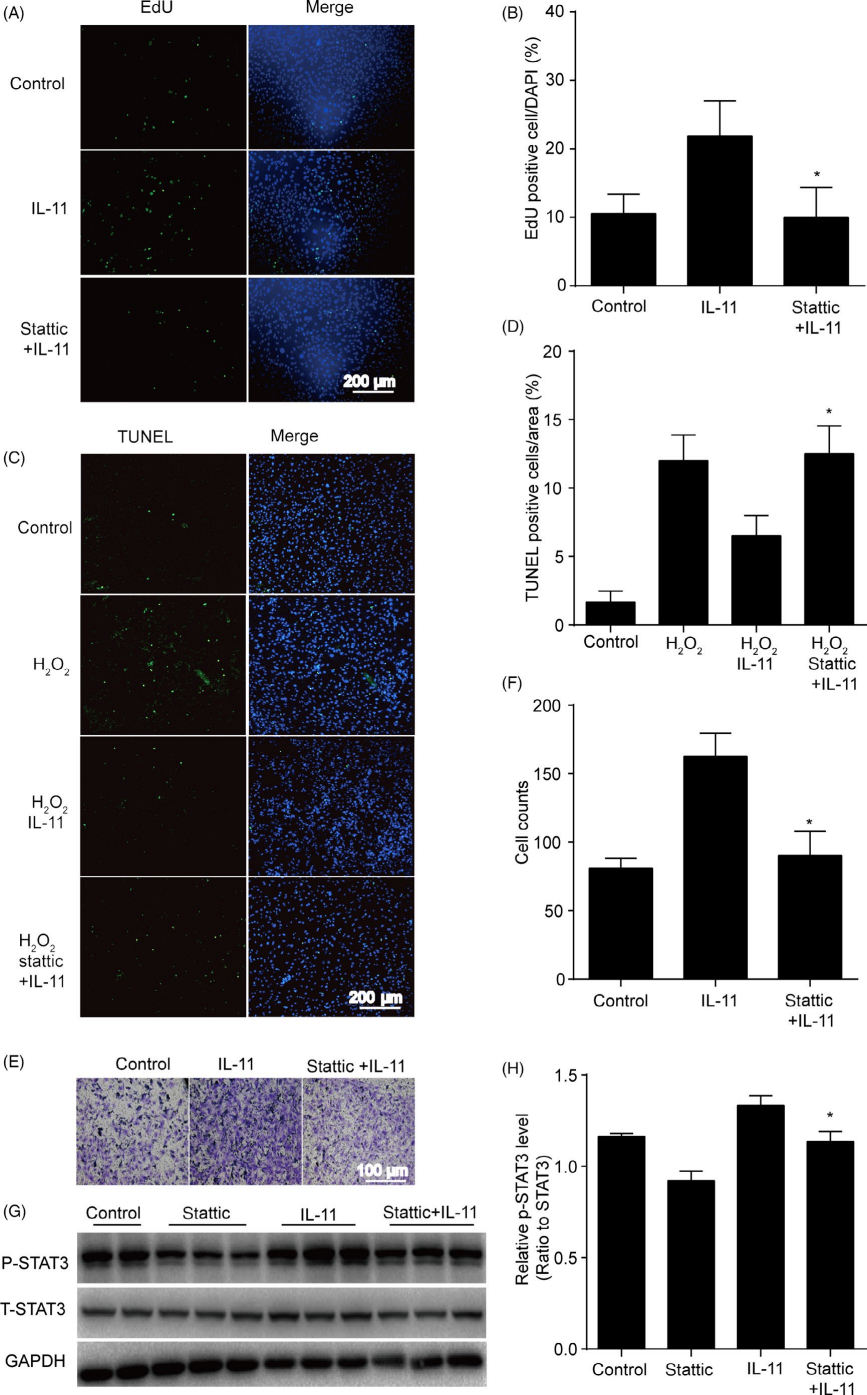
IL‐11 induced ADSCs proliferation, migration and anti‐apoptotic effects via STAT3 signalling pathway. A and B, Representative images and quantitative analysis of ADSCs proliferation. EdU assay was performed 24 h after IL‐11 (20 ng/mL) or IL‐11 + stattic (2 μmol/L) treatment. *P < .05 vs IL‐11. Two‐way ANOVA followed by Dunnett's post hoc test for statistical analyses; C and D, representative images and quantitative analysis of ADSCs apoptosis. TUNEL staining assay was performed 24 h in ADSCs after IL‐11 (20 ng/mL) or IL‐11 + stattic (2 μmol/L) treatment. H_2_O_2_: 200 μmol/L, 24 h. *P < .05 vs H_2_O_2_ + IL‐11. Two‐way ANOVA followed by Dunnett's post hoc test for statistical analyses; E and F, Cell migration analysis assessed by transwell assay 12 h in ADSCs with H_2_O_2_ after IL‐11 (20 ng/mL) or IL‐11 + stattic (2 μmol/L) treatment. *P < .05 vs IL‐11. Two‐way ANOVA followed by Dunnett's post hoc test for statistical analyses; G and H, Western blots and quantification protein expression of P‐STAT3 in ADSCs after IL‐11 (20 ng/mL) or IL‐11 + stattic (2 μmol/L) treatment. *P < .05 vs IL‐11. Unpaired Student's t test for statistical analyses; (n = 5 different fields, At least three independent experiments were performed)

To further confirm that the STAT3 signalling pathway was responsible for IL‐11‐induced ADSCs proliferation, migration and anti‐apoptotic effects, we used the inhibitor of the STAT3 (stattic) for the next experiments. The results revealed that ADSCs proliferation were decreased after IL‐11 and stattic co‐treatment compared with IL‐11 treatment alone (*P* < .05, Figure 4A,B). Meanwhile, co‐treatment of stattic attenuated the anti‐apoptotic effects of IL‐11 (*P* < .05, Figure 4C,D), impaired the migration capacity of ADSCs (*P* < .05, Figure 4E,F) and also decreased the phosphorylation of STAT3 in ADSCs compared with IL‐11 treatment alone (*P* < .05, Figure 4G,H).

### 3.5 IL‐11 induced ADSCs proliferation, migration and anti‐apoptotic effects via IL‐11 alpha receptor

**Figure 5:**
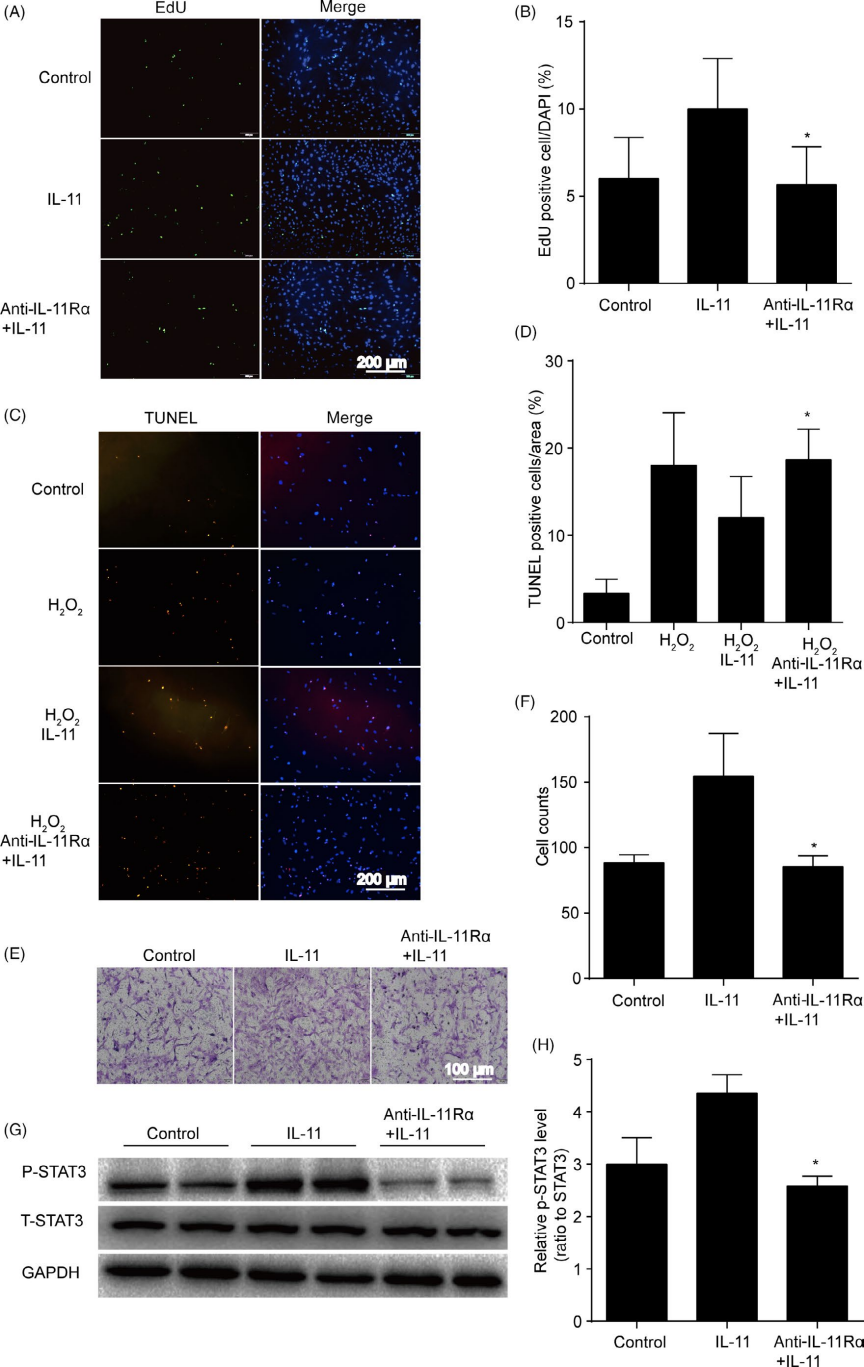
IL‐11 induced ADSCs proliferation, migration and anti‐apoptotic effects via IL‐11 alpha receptor. A and B, Representative images and quantitative analysis of ADSCs proliferation. EdU assay was performed 24 h after IL‐11 (20 ng/mL) or IL‐11 + anti‐IL‐11Rα (5 μg/mL) treatment. *P < .05 vs IL‐11. Two‐way ANOVA followed by Dunnett's post hoc test for statistical analyses; C and D, Representative images and quantitative analysis of ADSCs apoptosis. TUNEL staining assay was performed 24 h in ADSCs after IL‐11 (20 ng/mL) or IL‐11 + anti‐IL‐11Rα (5 μg/mL) treatment. H_2_O_2_: 200 μmol/L, 24 h. *P < .05 vs H_2_O_2_ + IL‐11. Two‐way ANOVA followed by Dunnett's post hoc test for statistical analyses; E and F, Cell migration analysis assessed by transwell assay 12 h in ADSCs with H_2_O_2_ after IL‐11 (20 ng/mL) or IL‐11 + anti‐IL‐11Rα (5 μg/mL) treatment. *P < .05 vs IL‐11. Two‐way ANOVA followed by Dunnett's post hoc test for statistical analyses; G and H, Western blots and quantification protein expression of P‐STAT3 in ADSCs after IL‐11 (20 ng/mL) or IL‐11 + anti‐IL‐11Rα (5 μg/mL) treatment. *P < .05 vs IL‐11. Unpaired Student's *t* test for statistical analyses; (n = 5 different fields, At least three independent experiments were performed)

The classic IL‐11 signalling is induced by binding to its alpha receptor, then IL‐11 alpha receptor (IL‐11Rα) interacted with membrane bound GP130 resulted in a trimeric complex. This trimer then associates with a second trimer to form a functional hexameric signalling complex.^31^ To explore whether IL‐11Rα was involved in IL‐11‐induced ADSCs proliferation, migration and anti‐apoptotic effects, we used the blocking antibody of IL‐11Rα for the next experiment. After co‐treatment with blocking antibody of IL‐11Rα, the effect of IL‐11 on ADSCs proliferation was diminished (*P* < .05, Figure 5A,B). Moreover, co‐treatment of IL‐11 and blocking antibody of IL‐11Rα attenuated the anti‐apoptotic effects of IL‐11 (*P* < .05, Figure 5C,D), impaired the migration capacity of ADSCs (*P* < .05, Figure 5E,F) and also decreased the phosphorylation of STAT3 in ADSCs compared with IL‐11 treatment alone (*P* < .05, Figure 5G,H).

### 3.6 IL‐11 enhanced the efficacy of ADSCs therapy in a mouse model of hindlimb ischaemia

To explore whether IL‐11 increased the therapeutic efficacy of ADSCs in treating ischaemic diseases, we established the IL‐11 overexpression ADSCs and implanted their into ischaemic mouse hindlimbs.

**Figure 6:**
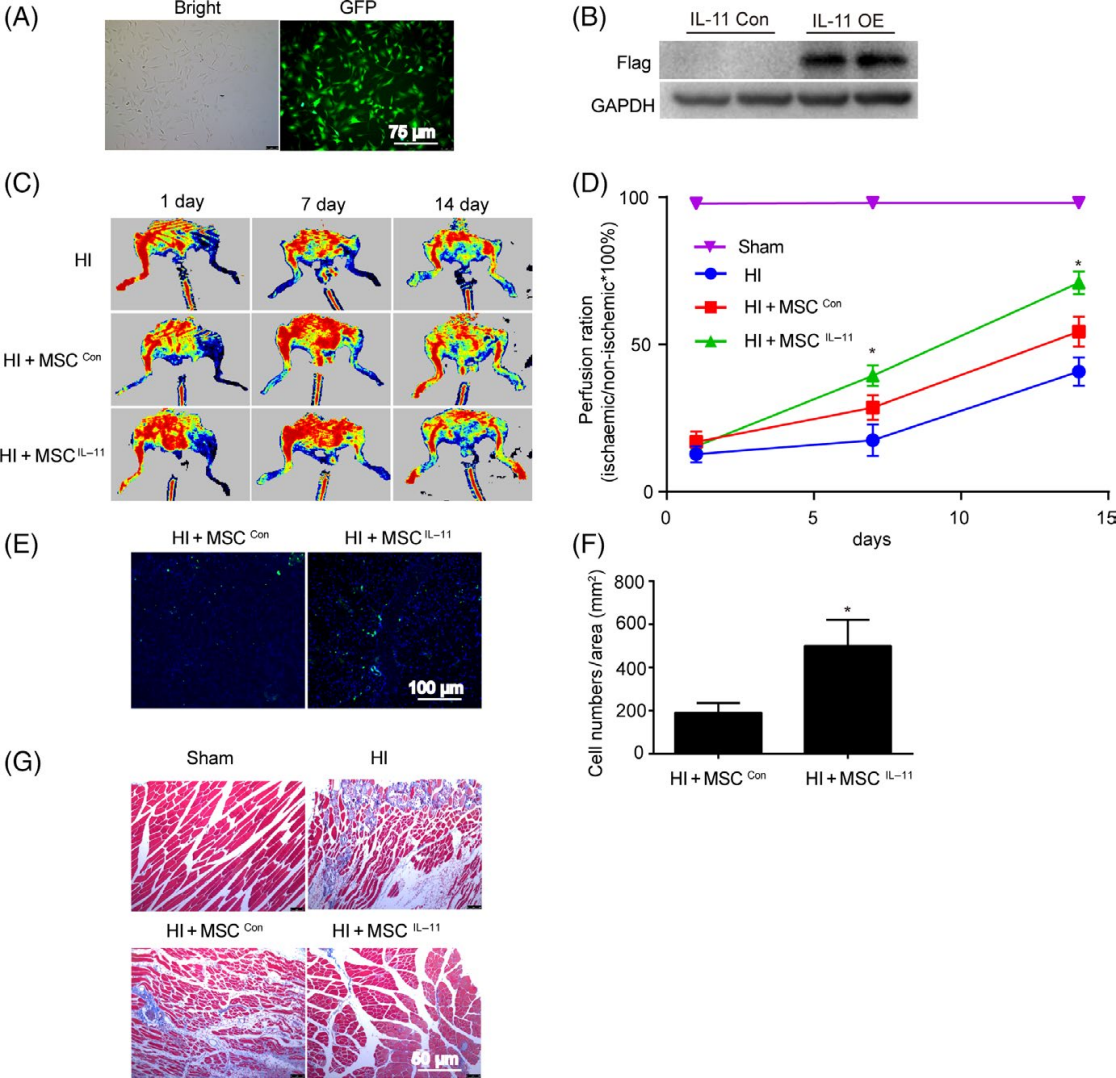
IL‐11 enhanced the efficacy of ADSCs therapy in a mouse model of hindlimb ischaemia. A and B, Expression of GFP fluorescence and the protein of flag in ADSCs after IL‐11 lentivirus transfection; C, representative blood perfusion images of mouse ischaemic hindlimbs after ADSCs transplantation; D, quantitative analysis of perfusion recovery by calculating the ischaemic/normal limb perfusion ratios. *P < .05 vs HI + MSC^con^. n = 6. Two‐way ANOVA followed by Dunnett's post hoc test for statistical analyses; E, representative images of ADSCs in ischaemic hindlimbs 14 days after transplantation. Muscle tissue was immunostained for GFP (green), DAPI (blue). F, Quantitative analysis of GFP‐positive cells in the ischaemic limbs by calculating the number of GFP‐positive cells per field. n = 3. *P < .05 vs HI + MSC^con^. Unpaired Student's *t* test for statistical analyses; G, representative images of Masson's trichrome staining of mouse ischaemic hindlimbs. Red represents viable muscle, and blue represents fibrosis

Expression of GFP fluorescence and the protein of flag confirmed successful construction of IL‐11 overexpressing ADSCs (Figure 6A,B). Laser Doppler perfusion image assay revealed that perfusion recovery in ischaemic limbs was significantly better in the IL‐11 overexpression ADSCs transplantation group than in the control group at both 7 and 14 days post‐therapy (day 7, 39.4 ± 8.6% vs 28.5 ± 10.3%; day 14, 71.0 ± 9.5% vs 54.4 ± 11.4%; *P* < .05, Figure 6C,D). The retention of implanted ADSCs was determined using histological analysis, and the overexpression of IL‐11 in ADSCs increased the ratio of cell survival in ischaemic tissues at 14 days post‐therapy (*P* < .05, Figure 6E,F). In addition, the fibrosis of ischaemic tissues was also significantly decreased after the IL‐11 overexpression ADSCs transplantation compared with implanted control ADSCs (Figure 6G). These results suggested that increased IL‐11 promoted the blood perfusion recovery and contributed to improve ADSCs engraftment in ischaemic tissues.

## 4 DISCUSSION

In the present study, we demonstrated that IL‐11 promoted ADSCs proliferation, migration and protected ADSCs from hydrogen peroxide‐induced cell apoptosis via STAT3 signalling pathway in vitro. Furthermore, we found that IL‐11 increased the retention of implanted ADSCs and perfusion recovery in ischaemic limbs. These findings indicate that IL‐11 may improve the therapeutic efficacy of ADSCs for the ischaemic diseases.

Adult stem cells including MSCs play key roles in the repair of damaged cells and the maintenance of tissue homeostasis.^32^ MSCs exist in most mammalian tissues and organs, such as the bone marrow, adipose tissue and umbilical cord. MSCs therapy has become a promising treatment for ischaemic disease, because of a large number of pre‐clinical evidence supporting their reparative potential.^4^, ^33^ However, poor survival of implanted MSCs and failure of stem cells engraftment in ischaemic environment occurs at early stages after delivery, raising a major challenge in the field.^33^ The extracellular matrices, autocrine and paracrine hormonal signals and tissues microenvironment can regulate the fate of stem cells.^2^ A comprehensive understanding of the mechanisms that enhance MSCs migration and survival in the injured tissues is critical for improving the repair capacity and therapeutic application of MSCs.

IL‐11 is produced by a variety of tissues including the heart, central nervous system and gastrointestinal tract.^15^ Because of the promotion of megakaryocytopoiesis and thrombopoiesis, recombinant human IL‐11 has been extensively used in patients for the prevention of chemotherapy‐induced thrombocytopenia.^21^ Previous study has suggested that the expression of IL‐11 mRNA and protein significantly decreases during cerebral ischaemia‐reperfusion injury.^25^ Analogous to previous report, we found that the expression of IL‐11 was decreased under hypoxia. We found that IL‐11 receptor was expressed in ADSCs. In addition, ADSCs viability was increased after IL‐11 treatment. IL‐11 also enhanced ADSCs viability under hypoxia. These results indicate that IL‐11 may play an important role under hypoxic conditions. Moreover, it has been found IL‐11 is a critical cytokine for the development of tumours in both the colon and stomach, which is mediated by promoting cell proliferation and reducing cell apoptosis.^34^, ^35^ In the colon and gastric cancer, the production of IL‐11 promotes cell migration through STAT3, which is involved in metastasis of tumour cells to other organs.^36^, ^37^ In addition, IL‐11 treatment reduces the cerebral infarct volume and improves neurological function, indicating that the neuroprotective ability of IL‐11 may be mediated by reducing neuronal apoptosis.^25^ Moreover, Aaron B. Waxman et al have demonstrated that IL‐11 diminishes oxidant‐mediated endothelial cell injury and exerts cytoprotective effects through STAT3 signal pathway.^24^ Indeed, promoting the proliferation and migrating of transplanted stem cells and reducing cell death in the ischaemic microenvironment are essential for improving the therapeutic efficacy of stem cells. In our study, the results showed that IL‐11 promoted ADSCs proliferation, migration and protected ADSCs from hydrogen peroxide‐induced cell apoptosis.

Furthermore, IL‐11 initiates signalling by binding to its alpha receptor, and then the IL‐11/IL‐11Rα complex interacts with GP130 as a tetrameric complex. The intracellular signalling pathways can be initiated following formation of this tetrameric complex. Previous studies have shown that IL‐11 regulates cellular function mainly through three signalling pathways including JAK‐STAT3, RAS‐RAF‐ERK and PI3K‐AKT‐mTORC1 pathway,^22^, ^31^ which are the downstream of IL‐11Rα signalling. Thus, we examined the expression of P‐STAT3, P‐ERK1/2 and P‐mTOR in ADSCs to determine the molecular mechanisms underlying the effects of IL‐11 upon ADSCs function. We found that the expression of P‐STAT3 was significantly up‐regulated after IL‐11 treatment in ADSCs. These findings suggested that IL‐11 may regulate the function of ADSCs through IL‐11‐IL‐11Rα‐STAT3 signalling pathway. Indeed, STAT3 regulates the transcription of target genes that determined various important biological functions. The phosphorylation of STAT3 leads to expression of pluripotency genes which is responsible for self‐renewal and the undifferentiated state of mouse embryonic stem cells.^38^, ^39^ In addition, the activation of STAT3 promotes human adult stem cells migration and survival and plays critical roles in the proliferation of adult stem cells.^40^, ^41^, ^42^ To further confirm our hypothesis, the inhibitor of the STAT3 (stattic) and blocking antibody of IL‐11Rα was used in our study. These results showed that both stattic and the blocking antibody of IL‐11Rα impaired IL‐11‐induced ADSCs proliferation, migration and anti‐apoptotic effects.

The poor survival of implanted MSCs in the ischaemic environment is the main impediment of the therapeutic potential of MSCs, so it is very important to explore novel ways to enhance the survival of transplanted MSCs. We found that IL‐11 improved perfusion recovery and increased the retention of implanted ADSCs in ischaemic limbs. Furthermore, previous research has shown that IL‐11 can promote the mobilization of CD34^+^/VEGFR2^+^ cells, induce collateral vessel regeneration and improved perfusion recovery in ischaemic limbs.^43^ In our study, the increasing blood perfusion may also be attributed to the direct effects of IL‐11. In addition, the fibrosis of ischaemic tissues was also significantly decreased after transplantation of IL‐11 overexpression ADSCs. These findings suggested that IL‐11 can improve the efficacy of stem cell therapy. Our study firstly identifies the IL‐11 cytoprotective effect to transplanted ADSCs, provide a new hint to interfere IL‐11 in order to improve the MSC transplantation therapy outcome.

In summary, we demonstrate that IL‐11 induces ADSCs proliferation, migration and anti‐apoptotic effects via STAT3 signalling pathway and acts as a cytoprotective factor regulating ADSCs engraftment in ischaemic tissues.

## CONFLICT OF INTEREST

The authors declare no conflict of interest.

## AUTHOR CONTRIBUTIONS

WLY and TTO performed the study. HJ and DLJ contributed to data acquisition and analysis. ZYQ and TTO contributed to the figures and statistical analysis. WLY and SNZ drafted the manuscript. YZZ and JYQ revised the manuscript. JBG, SNZ and AJS conceived and designed the study. All authors read and approved the final manuscript.

## Data Availability

The data that support the findings of this study are available from the corresponding author upon reasonable request.
