# Supplementary figures and images for: Interleukin‐11 regulates the fate of adipose‐derived mesenchymal stem cells via STAT3 signalling pathways
Source: Cell Prolif. 2020 Apr 9;53(5):e12771. doi: 10.1111/cpr.12771 (PMC7260062; doi:10.1111/cpr.12771)

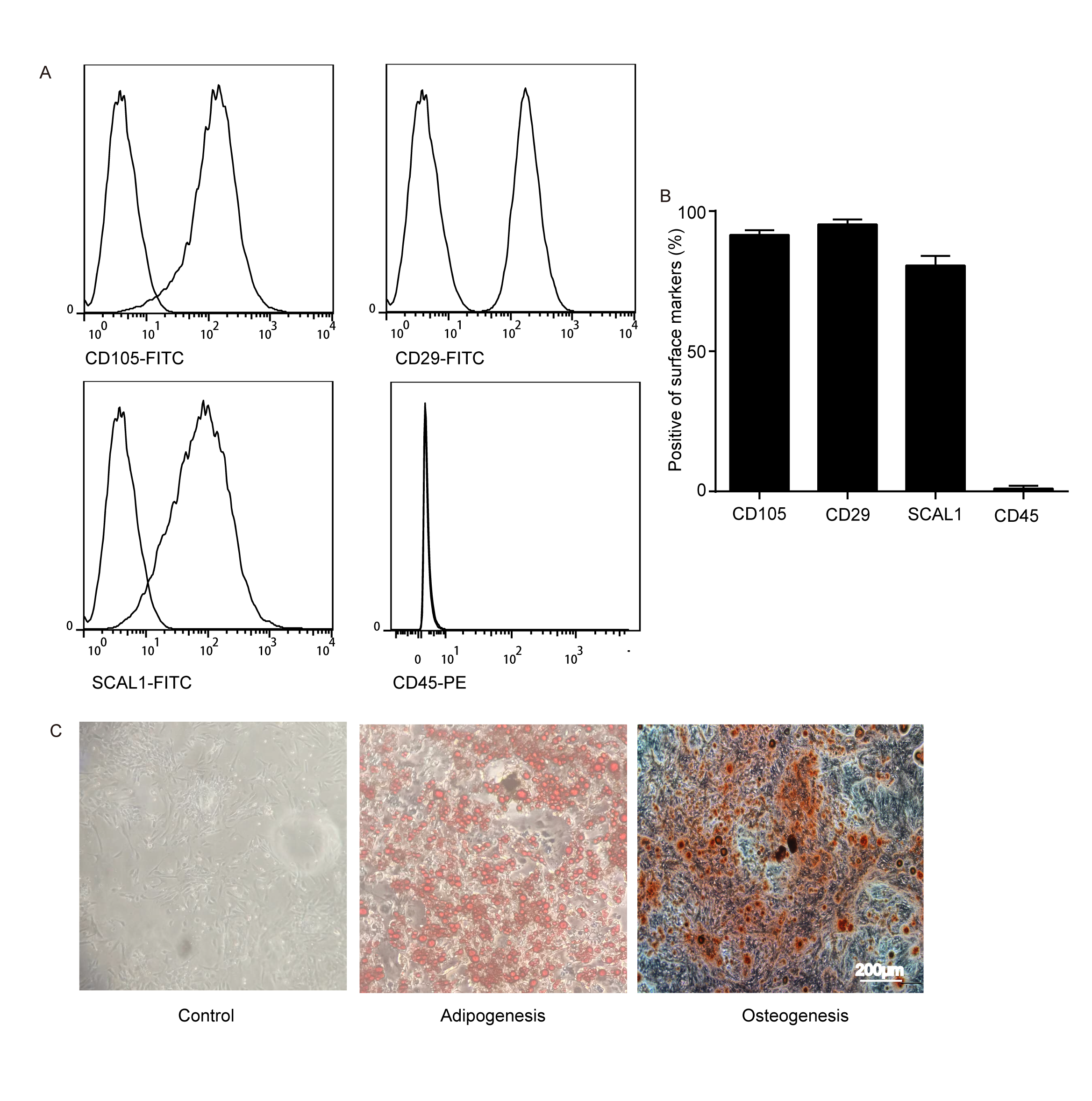

Supplement: Supplementary file 1 [file CPR-53-e12771-s001.tif]

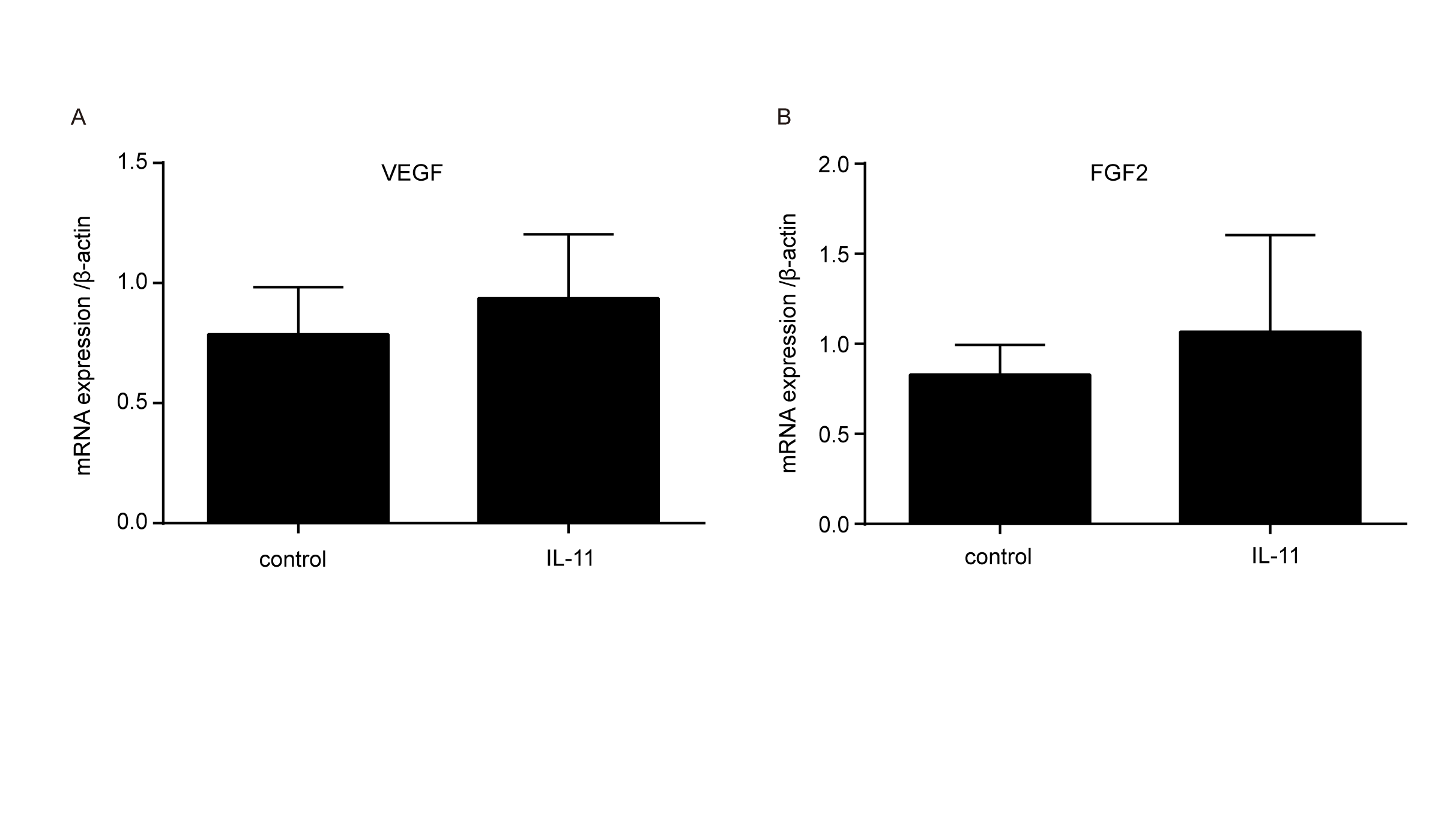

Supplement: Supplementary file 2 [file CPR-53-e12771-s002.tif]
